# Kinetic Analysis of Freeze-Thaw Stability of Mayonnaise

**DOI:** 10.3390/foods7050075

**Published:** 2018-05-08

**Authors:** Islam Muhammad Shariful, Nakako Katsuno, Takahisa Nishizu

**Affiliations:** Department of Applied Life Science, Gifu University, Yanagido1-1, Gifu 501-1193, Japan; mobas14@yahoo.com (I.M.S.); nkatsuno@gifu-u.ac.jp (N.K.)

**Keywords:** mayonnaise, emulsion, freeze-thaw, destabilization, crystallization, kinetic parameters

## Abstract

Kinetic analysis was used to study the destabilization of mayonnaise by focusing on the fat crystals. Mayonnaise prepared from rapeseed oil and soybean oil was stored at temperatures ranging from −20 to −40 °C. The destabilization kinetic parameters were measured by observing oil separation over time. The destabilization rate constant, *k_d_*, increased with decreasing temperature. The highest value of *k_d_* was 1.28 × 10^−3^ min^−1^ at −40 °C for rapeseed oil mayonnaise (RoM) and the lowest was 1.95 × 10^−6^ min^−1^ at −20 °C for soybean oil mayonnaise (SoM). At each temperature, the *k_d_* value in RoM was higher than that in SoM. However, the order of destabilization, *n*, followed no specific pattern. The crystallization rate constant, *K_c_*, and Avrami constant, *n*, were calculated using microscopic images of the fat crystals. The increase in crystallization kinetic parameters with decreasing temperature revealed changes in crystal behavior. Both the destabilization rate constant, *k_d_*, and the crystallization rate constant, *K_c_*, depended on the temperature. This temperature dependency behavior showed a correlation between *k_d_* and *K_c_*, suggesting that the destabilization rate depended on the rate of growth of fat crystals during the freeze-thawing of mayonnaise.

## 1. Introduction

Mayonnaise is a semi-solid oil-in-water (O/W) emulsion composed of vegetable oil as the oil phase and vinegar, egg yolk (emulsifier), salt, and sugar as the water phase. Mayonnaise usually contains 70–80% fat [1] and is widely consumed with different foods to improve their palatability and desirability. Many food emulsions, such as sauces and beverages, can be frozen to increase their shelf life [2,3,4] or are frozen for easier commercial transportation. However, the destabilization or phase separation of emulsions during freeze-thawing is an important problem to overcome.

Most O/W emulsions are destabilized after freeze-thawing because of crystallization of the fat and water in the respective phases [2,5] through coalescence or partial coalescence [6]. Several approaches have been reported that study how water and fat crystallization affects destabilization during the freeze-thawing of O/W emulsions. Ice crystals can destabilize emulsions by flocculating oil droplets, increasing ionic strength, and changing the pH in the unfrozen aqueous phase [5,7,8]. These changes in the emulsion increase contact between oil droplets. Ice crystals can also become larger by recrystallizing during storage, which disrupts the interfacial membranes [9]. Ice crystal may have influence on destabilization in mayonnaise type emulsion but fat crystal is strongly related with the stability during freeze-thawing [10]. During freezing, fat crystals can penetrate neighboring oil droplets, which causes partial coalescence in the O/W emulsion [11,12]. These partially-coalesced droplets then collapse to form larger droplets, which appear in the separated oil when the emulsions are heated. Research interest in the freeze-thaw stability of mayonnaise-type O/W emulsions has been increasing. Recently, the role of fat crystals on the freeze-thaw stability of mayonnaise type emulsions has been reported. Magnusson et al. [3] reported that the oil composition and freezing rate significantly affect freeze-thaw stability and that a high oleic acid content in the oil is negatively correlated with the freeze-thaw stability of mayonnaise. Miyagawa et al. [10] also reported similar results, finding that the time for crystallization of soybean oil was longer than that of rapeseed oil, suggesting that the dispersion stability of mayonnaise depended on the crystallization of the oil. In canola oil, Ishibashi et al. [13] observed the partial coalescence of oil droplets by fat crystals. Based on these studies, it could be assumed that the freeze-thaw stability of mayonnaise would depend on the amount of fat crystal growth. Fat crystals can increase coalescence by increasing the number of effective collisions between droplets and higher and faster crystal growth would result in poor stability. However, the influence of crystal amount (nucleation and growth) on destabilization is unclear. Basically, the phenomena of destabilization and crystallization are different. Destabilization is the consequence of crystallization so making it difficult to evaluate these two phenomena simultaneously. 

To clarify the influence of crystallization on destabilization, kinetic analysis would be an effective tool. For this reason, the effect of temperature on the rates of destabilization and crystallization must be considered. A typical first order rate equation [14] has been used to analyze destabilization kinetics and crystallization kinetics using the Avrami model [15]. No quantitative approach has yet been reported for analyzing the destabilization and crystallization of mayonnaise. Therefore, the objective of the present study is to clarify the destabilization and crystallization kinetic parameters in mayonnaise and so improve the understanding of the influence of crystallization on destabilization.

## 2. Materials and Methods

### 2.1. Materials

The ingredients of the oil and water phases for the mayonnaise were obtained from the Oriental Yeast Company (Tokyo, Japan). Two types of mayonnaise were prepared, rapeseed oil mayonnaise (RoM) and soybean oil mayonnaise (SoM). The oil phase consisted of only rapeseed oil or soybean oil and the water phase consisted of egg yolk as the emulsifier, salt, sugar, water and vinegar. Table 1 and Table 2 show the composition of the water and oil phases of the two mayonnaises and fatty acid composition of oil phases, respectively. The principal fatty acid in rapeseed oil was oleic acid (18:1) and in soybean oil it was linoleic acid (18:2). 

### 2.2. Methodology

#### 2.2.1. Preparation of Mayonnaise Samples

To prepare 32.6 g of mayonnaise, 22.8 g of the oil phase and 9.8 g of the water phase were used at a ratio of 7:3. The water phase was placed in a 50-mL centrifuge tube then the oil phase was added and mixed in eight stages. Each time, one eighth of the oil phase was added then homogenized for 45 s at 25,000 rpm concluding with homogenization for 1 min [16]. A Polytron Pt 1200e homogenizer (Kinematica AG, Lucerne, Switzerland) was used for preparing the mayonnaise. The median diameter of the oil droplets was 1.1 μm, which was been calculated from the microscopic image of oil droplets through image processing using Image J software (Image J 1.43u, Java 1.6.0_10 (32-bit), National Institute of Health, Bethesda, MD, USA).

#### 2.2.2. Measurement of Freeze-Thaw Stability of Mayonnaise Using a Capillary Tube

The freeze-thaw stability of the mayonnaise samples was tested using a capillary tube (Drummond Scientific Co., Broomall, PA, USA). The volume of the capillary was 6.6 μL, with an inner diameter of 0.34 mm, an outer diameter of 0.86 mm and a length of 72 mm. The sample was placed in the tube using a suction pump (Rikakikai Co., Ltd., Tokyo, Japan). Araldite adhesive was used to seal the opening of the capillary tube. The capillary tubes with samples were then frozen directly by hanging them in a chilled liquid at temperatures ranging from −20 to −40 °C. The oil separation had been measured with time until the separated oil fraction was reached at 95%. After freezing, the samples were thawed in a hot water bath at 60 °C for 5 min then centrifuged for 5 min at 1250× *g* in a KN-70 table top centrifuge (Kubota Corporation, Tokyo, Japan). After centrifugation the separated oil formed a distinct layer in the capillary. Images of the capillary with the separated oil were taken using a camera (Canon Speedlite 90EX, EOS, Canon Inc., Tokyo, Japan) and then used to calculate the weight of the separated oil using Image J software (Image J 1.43u, Java 1.6.0_10 (32-bit), National Institute of Health, Bethesda, MD, USA).

#### 2.2.3. Polarized Light Microscopy

To calculate the crystallization kinetic parameters, the fat crystals in the mayonnaise samples were observed during storage at −20, −30 and −40 °C, using a polarized light microscope (PLM) (DP73, Olympus, Tokyo, Japan). The samples were placed in a temperature-controlled microscope stage (LK-600PM and 10021, Linkam Scientific Instruments Ltd., Tadworth, UK). The samples were placed between two cover slips then frozen to temperatures of −20, −30 and −40 °C at a rate of 5 °C/min then held for 10 h, 8 h and 5 h at −20, −30 and −40 °C, respectively, for the rapeseed oil mayonnaise. The soybean oil mayonnaise samples were held for 24 h, 18 h and 10 h at −20, −30 and −40 °C, respectively. Images were captured from the first appearance of fat crystals. The sample chamber of the microscope stage was filled with nitrogen gas to prevent water condensation on the surface of the samples [13]. Changes in the amount of fat crystals (crystallized area) with time were measured by image processing using Image J software, which was also used to calculate the crystallization kinetic parameters.

#### 2.2.4. Thermal Behaviors of Mayonnaise and Oil Phases Using Differential Scanning Calorimetry (DSC)

A heat-flux type Exstar SII-6200 DSC (Seiko Instruments Inc., Chiba, Japan) was used to investigate the thermal behavior of the mayonnaise, and oil phases. The samples (10 mg) were placed in an aluminum pan and then sealed tightly. An empty aluminum pan was used as reference. A temperature profile from 0 to −75 °C, holding for 5 min at a cooling or heating rate of 5 °C/ min was used. The crystallization temperature was determined from the onset temperature of the peaks and the melting temperature from the peak top temperature on the thermograms.

#### 2.2.5. Destabilization Kinetic Analysis

Civan et al. [14] suggested that the destabilization of emulsions was a single irreversible isothermal reaction. Thus, the destabilization of mayonnaise can be written as Equation (1):

(1) $$-\frac{dS_{m}}{dt}\;=\;k_{d}S_{m}{}^{n}$$

where *S_m_* is the remaining oil fraction of the mayonnaise, *k_d_* is the destabilization rate constant, *n* is the order of destabilization, and *t* time.

Taking this into account, the initiation of mayonnaise destabilization during freezing is delayed by a certain time (induction time), *t_i_*, and stability at that time, *S_i_*, so the initial condition can be defined by Equation (2):

*S_m_* = *S_i_*, *t* = *t_i_* (2)

Therefore, the analytical solution of Equation (1) under isothermal conditions subject to Equation (2) can be given as Equation (3):

(3) $$S_{m}{}^{1-n}\;=\;S_{i}{}^{1-n}\;-\;\left(1-n\right)k_{d}\left(t-t_{i}\right)$$

Thus, determining the destabilization rate constant, *k_d_*, and the order of destabilization, *n*, could be achieved using differential method of analysis from the fitted graph using Equation (3) [17]. 

#### 2.2.6. Crystallization Kinetic Analysis

The Avrami equation has been used for the kinetic analysis of crystallization [15] (Equation (4)):

(4) $$1-X_{t}\;=\;\exp\;\left(-K_{c}t^{n}\right)$$

where *X_t_* is the relative degree of crystallinity at time *t*; *K_c_* is the crystallization rate constant, which depends on temperature; and *n* is the Avrami exponent.

#### 2.2.7. Statistical Analysis

All experiments were repeated four times. The differences between means (from replicates) were examined using Tukey’s honest significant difference test at a significance level of *p* < 0.05 using Kaleida Graph (Synergy Software, Reading, PA, USA).

## 3. Results

### 3.1. Freeze-Thaw Stability of Mayonnaise at Different Freezing Temperatures

**Figure 1:**
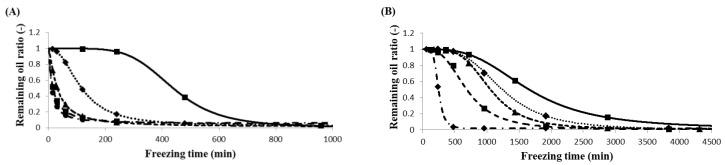
The stability of mayonnaise showing remaining oil ratio versus time after fitting the data. (**A**) RoM (rapeseed oil mayonnaise); and (**B**) SoM (soybean oil mayonnaise) for freezing at temperatures of –20 °C (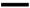), –25 °C (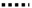), –30 °C (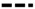), –35 °C (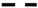), and – 40 °C (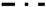). Points on the fitted curves indicate the experimental data. Results are expressed as the mean of four trials.

The oil separation after freezing at different temperatures and times differed significantly, as shown in Figure 1A,B for RoM and SoM, respectively. The graphs show a typical sigmoidal pattern: first, the preparation for oil separation (induction time), then a sudden increase in the speed of separation and, finally, the separation slowed down. The point when the remaining oil ratio reached 0.95 was considered the induction time. The induction times of oil separation for RoM and SoM are shown in Table 3. Mayonnaise kept at temperature 0 °C was not frozen and did not separate, even after storage for seven days.

The longest induction time at −20 °C for SoM was 633.3 min and the shortest 0.9 min at −40 °C for RoM. The induction time varied significantly with temperature and oil type, and was lower for RoM than SoM at each freezing temperature. When considering the time for kinetic measurements, the induction time was ignored because, during this period, the rate of separation was zero.

After the induction period, the speed of oil separation increased. It also varied with temperature and between RoM and SoM. The time needed to reach the maximum separated fraction was lower for RoM than SoM and decreased as the temperature decreased from −20 °C to −40 °C. The mayonnaise samples frozen at −20 °C took the longest time to reach their maximum separated fraction. Overall, the rate of oil separation was influenced by the temperature and the oil type. 

### 3.2. Destabilization Kinetic Parameters Due to Temperature Change

Table 4 shows the values of the kinetic parameters at different temperatures for RoM and SoM. The destabilization rate constant, *k_d_*, had the lowest value of 1.95 × 10^−6^ min^−1^ at −20 °C for SoM and the highest value of 1.28 × 10^−3^ min^−1^ for RoM at −40 °C. The *k_d_* value for RoM was higher than SoM at each temperature and increased with decreasing temperatures. Therefore, for the particle sizes and other experimental conditions used in the present study, it can be concluded that the main physical condition causing variation in the destabilization rate constant was the temperature. 

The order of destabilization, *n*, followed no specific pattern with regard to temperature for both RoM and SoM. The values ranged from 0.05 to 1.75 for RoM and 0.11 to 1.35 for SoM. The *k_d_* value with unified *n* value of 1 also increased with decreasing temperature.

### 3.3. Crystallization Kinetics Due to Temperature Change

**Figure 2:**
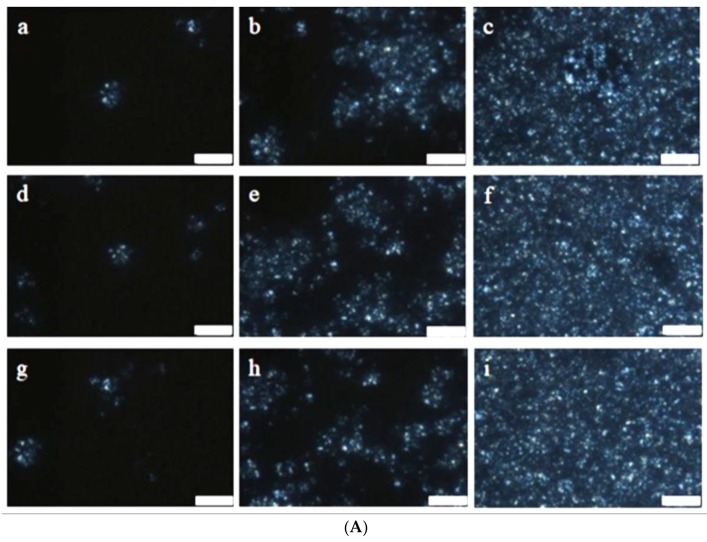

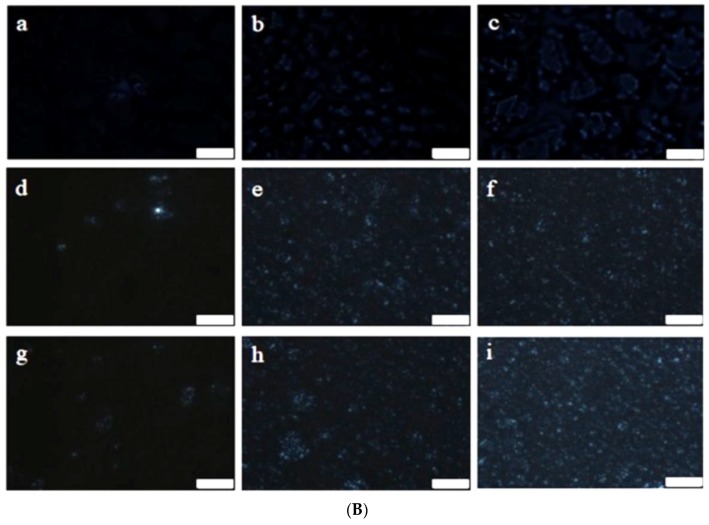
(**A**) Microscopic images of RoM fat crystals (white dots) using PLM (polarized light microscope) Images: (**a**–**c**) storage at −20 °C for 6 h, 6.5 h and 7 h, respectively; (**d**–**f**) storage at −30 °C for 2 h, 2.5 h min and 3 h, respectively; and (**g**–**i**) storage at −40 °C for 50 min, 1.5 h and 3 h, respectively. Scale bar is 20 μm. (**B**) Microscopic images of SoM fat crystals (white dots) using PLM: (**a**–**c**) storage at −20 °C for 14 h 20 min, 15 h 50 min and 16 h 35 min, respectively; (**d**–**f**) storage at −30 °C for 11 h 50 min, 12 h 50 min and 13 h 20 min, respectively; and (**g**–**i**) storage at −40 °C for 4 h 20 min, 4 h 50 min and 5 h 5 min respectively. Scale bar is 20 μm.

The images of fat crystals in RoM and SoM (Figure 2A,B) show that the growth of fat crystals in RoM was higher than in SoM. The differences in crystal growth were governed by temperature and the type of oil used. In RoM, oleic acid was the dominant fatty acid and in SoM it was linoleic acid so the crystal growth was faster in RoM than in SoM. To quantify the differences in the crystallization behaviors in RoM and SoM at different temperatures, the fat crystal data from the PLM images (Figure 2A,B) were fitted separately using the Avrami equation for crystallization kinetic analysis.

The induction times for fat crystal generation are shown in Table 5, and the crystallization rate constant, *K_c_*, and Avrami exponent, *n*, are shown in Table 6. The Avrami exponent, *n*, expresses the change in crystal dimensions with changes in temperature. In the present study, both RoM and SoM exhibited a higher Avrami exponent, *n*, with decreasing temperature, which means that the dimensions of the fat crystals changed as the temperature changed. 

The differences between RoM and SoM in the *n* value observed at each temperature were not significant. The crystallization rate constant, *K_c_*, indicating the influence of temperature on the nucleation rate of fat crystals increased with decreasing temperature.

The highest nucleation rate was found at −40 °C for RoM with a value of 1.14 × 10^−2^ min^−^¹ and the lowest one for SoM at −20 °C with a value of 7.87 × 10^−5^ min^−^¹. However, because of the variation in the *n* value, the unit of *K_c_* would also differ.

Therefore, to examine the effect of temperature and oil type, *K_c_* was recalculated using a unified *n* value of 1 because the Avrami exponent *n* for temperature and oil type was close to 1. The recalculated *K_c_* values are shown in Table 7. 

The recalculated *K_c_* values (Table 7) also show the same tendency as those in Table 6, i.e., with decreasing temperature, the *K_c_* value increased and was higher in RoM than in SoM. Similar to the destabilization rate constant, *k_d_*, the crystallization rate constant, *K_c_*, also depended on temperature.

### 3.4. Influence of Crystallization on Destabilization

**Figure 3:**
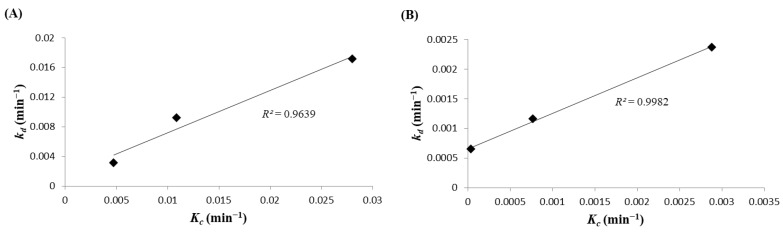
Relationship between crystallization rate constant *K_c_* and destabilization rate constant *k_d_* for: (**A**) rapeseed oil mayonnaise; and (**B**) soybean oil mayonnaise.

Figure 3A,B shows the relationship between the crystallization rate constant, *K_c_*, and the destabilization rate constant, *k_d_*, of RoM and SoM, respectively. For both types of mayonnaise, there were strong correlations between *k_d_* and *K_c_*, with *R*^2^ values of 0.96 and 0.99 for RoM and SoM, respectively. These results show that both parameters were strongly dependent on temperature and increased with decreasing temperature. The increasing *K_c_* value at a lower temperature indicated that changes in the nucleation and/or growth rates of the fat crystals might influence the separation rate. 

### 3.5. Thermal Behavior of Mayonnaise

**Figure 4:**
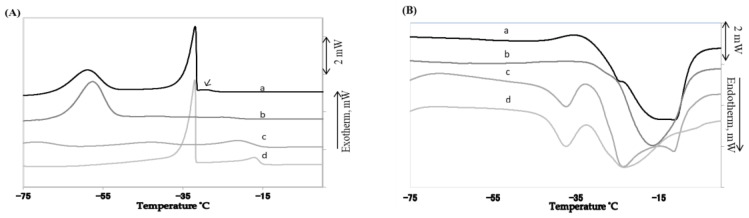
(**A**). Thermal behavior with DSC during cooling at a rate of 5 °C/min: (a) RoM; (b) rapeseed oil; (c) soybean oil; and (d) SoM from 0 to −75 °C. (**B**) Thermal behavior with DSC (Differential Scanning Calorimetry) during melting at a rate of 5 °C/min: (a) rapeseed oil; (b) RoM; (c) SoM; and (d) soybean oil from −75 to 0 °C.

The thermal behavior of the two types of mayonnaise and their oil phases was investigated using DSC. The DSC thermograms of crystallization and melting of both RoM and SoM and their oil phases are shown in Figure 4A during cooling from 0 to −75 °C at a rate of 5 °C/min and Figure 4B during melting from −75 to 0 °C. The RoM exhibited three exothermic peaks during cooling. The sharp, long exothermic peak at −31.8 °C indicated the crystallization of water in the mayonnaise [3]. The small exothermic peak (marked by an arrow in Figure 4A before water crystallization at −29 °C) indicated the crystallization of high melting saturated fat and another exothermic peak after water crystallization at −58.1 °C indicated the crystallization of the low melting unsaturated fat fraction of mayonnaise. The exothermic peaks for rapeseed oil also indicated the crystallization of low melting unsaturated fat. The exothermic peaks for saturated fat and water were distinct for SoM at −17.6 °C and −31.8 °C, respectively. The broad exothermic peaks at −70.8 °C and −53.3 °C indicated unsaturated fat and the peak at −21.6 °C indicated the saturated fat of soybean oil. These DSC results indicated that in both mayonnaise samples the high melting saturated fat fraction crystallized first, followed by the water and then the low melting unsaturated fat [13].

The DSC thermograms of mayonnaise and their oil phases during melting from −75 to 0 °C are shown in Figure 4B. The endothermic peaks at −10 °C indicated the melting of ice in both mayonnaise samples. The endothermic peaks at −24.7 and −15.7 °C for rapeseed oil and RoM, respectively, corresponded to the melting of unsaturated fat. The broad endothermic peaks at −37.4 and −23.9 °C for soybean oil and SoM, respectively, corresponded to the melting of unsaturated fat. These results show that the crystallinity of RoM and SoM differed during freezing from −20 to −40 °C. This difference might have caused the differences in the crystallization rate constant, *K_c_*.

## 4. Discussion

The kinetics of destabilization and crystallization for crystallization temperatures ranging from −20 to −40 °C and for two oil types were analyzed. Two different types of oil, rapeseed and soybean, were used to provide compositional variation. The variation of temperatures and oil types led to differences in induction times as well as in the kinetic parameters. The induction time for oil separation decreased with decreasing temperature and was lower in RoM than in SoM at each temperature (Table 3). In the present study, the stability of mayonnaise was evaluated using capillary tubes by calculating the induction time or oil separation and the kinetic parameters. The induction time for fat crystal generation was also calculated from microscopic images. The induction time for oil separation (Table 3) and induction time for fat crystal generation (Table 5) showed similar trends with temperature, although they did not overlap each other. This might have been due to differences in the size of the samples. The lowest values of induction time for oil separation were measured using capillary tubes rather than larger bulky tubes. The induction time for oil separation and induction time for fat crystal generation would be the same if the sample size and thickness was the same as the microscopic sample but, practically, it is difficult to measure oil separation using microscopic samples. These results suggest that the oil separation started with the generation of fat crystals.

After the induction period, the oil started to separate. Figure 1 shows that the speed of oil separation depended on the temperature as well as the oil type. The highest speed of oil separation occurred at −40 °C and the lowest at −20 °C for both types of mayonnaises. The fastest speed of oil separation led to the highest destabilization rate constant, *k_d_*, at −40 °C and decreased with increasing temperature. On the other hand, the microscopic images of fat crystals in Figure 2 showed that speed of the growth of fat crystals was higher at −40 °C than at −20 °C and also faster in RoM than in SoM. The kinetic analysis of crystallization in Table 6 showed that the crystallization rate constant, *K_c_*, increased with decreasing temperature. The increasing *K_c_* value indicated changes in the nucleation and/or growth rate of the fat crystals. Because *K_c_* is a combined function of nucleation and growth of the fat crystal, it also indicates changes in crystal morphology. Another parameter, the Avrami exponent, *n*, also increased with decreasing temperature. This could indicate differences in the geometry of crystal growth and the type of nucleation because *n* is a function of the number of dimensions.

The DSC results shown in Figure 4A,B confirmed that the crystallization temperature of rapeseed oil and soybean oil differed. These differences in crystallization resulted from the lower *K_c_* value for SoM compared with RoM. However, the amount of fat crystals depends on the fatty acid composition of the oil phase. The rapeseed oil was mainly composed of oleic acid, whereas, in soybean oil, linoleic acid was dominant [18]. The melting point of oleic acid is higher than that of linoleic acid. These compositional variations resulted in the higher quantity of fat crystals in RoM than in SoM that produced a higher value of *k_d_* in RoM than in SoM at each temperature. Magnusson et al. [3] have reported that a high content of oleic acid negatively affected the freeze-thaw stability of a mayonnaise-type O/W emulsion. Figure 3A,B show an almost linear positive relationship between crystal growth and oil separation and also show that both parameters increased with decreasing temperature. The tendency for the destabilization rate to increase with decreasing temperature might have resulted from the crystallization of the fat. The PLM image in Figure 2 showed that the growth of fat crystals was faster with decreasing temperature which agreed with results reported by Magnusson et al. [3]. They found that the crystallization of fat and water mostly influenced the freeze-thaw stability of O/W emulsions. Both *k_d_* and *K_c_* were the coincidence isothermal freeze-storage. The increasing quantity of fat crystals with decreasing temperature was caused by supersaturation at lower temperatures. In the shorter time, higher amounts of fat crystal were generated at the lower temperature resulting in the higher value of *k_d_*. This might have been caused by a high collision frequency between oil droplets causing a higher rate of coalescence.

The destabilization order, *n*, followed no specific pattern which meant that the mechanism of coalescence differed at each temperature. Recently, Ishibashi et al. [13] reported that canola and soybean oil mayonnaise destabilize in different ways and that the mechanism of coalescence also differed from −15 to −20 and −30 °C because of morphological changes in the fat crystals. In the crystallization kinetics, the Avrami exponent, *n*, was found to increase with decreasing temperature which showed that the change in crystal type occurred with decreasing temperature. In both mayonnaise samples, the value of the Avrami constant increased by 0.7 from −20 °C to −40 °C resulting in a change of crystal type from needle to plate. Although there was no direct clear evidence for this explanation, Ishibashi et al. [13] observed changes in crystal morphology and polymorphic change using X-ray diffraction (XRD). Kaufmann et al., Kalnin and Silva et al. [19,20,21] also reported changes in the form and structure of fat crystals with temperature change. The changes in crystal types occurring in RoM and SoM were insignificant, but the change in crystallization rate differed significantly. The present study has suggested that crystallization is the key factor in destabilization and that the speed of growth of crystals greatly affects destabilization. 

## 5. Conclusions

In this study, the freeze-thaw stability of RoM and SoM was investigated kinetically to clarify the influence of fat crystals on destabilization. The induction time for oil separation and fat crystal generation decreased with decreasing temperature, thus supporting the linking of oil separation to fat crystal generation. Both the destabilization rate constant, *k_d_*, and crystallization rate constant, *K_c_*, increased with decreasing temperature, showing the temperature dependency which revealed the dependency of *k_d_* on *K_c_*. The destabilization of mayonnaise during freezing was influenced by the crystallization properties of the fat with its stability being influenced mostly by the growth of fat crystals rather than the type of crystal. The longer induction time and lower crystal growth of SoM gave it a greater stability than RoM. 

## Figures and Tables

**Table 1:** Ingredients of the two mayonnaises (prepared in batches of 32.6 g).

Ingredient	% Weight	Weight (g)
Water phase		
Egg yolk	14.1	4.6
Vinegar	7.7	2.5
Egg Albumen	3.7	1.2
Salt	2.1	0.7
Sugar	0.3	0.1
Water	2.1	0.7
Total	30	9.8
Oil phase		
Rapeseed oil/Soybean oil	70	22.8

**Table 2:** Fatty acid composition of oil phases.

Oil	Fatty Acid (%)		
	Oleic Acid (18:1)	Linoleic Acid (18:2)	Linolenic Acid (18:3)
Rapeseed	72	13	4
Soybean	18	65	2

**Table 3:** Induction time of oil separation for RoM and SoM.

Temperature (°C)	Induction Time (min)	
	RoM	SoM
−20	251.9 ^a^	633.3 ^a^
−25	33.2 ^b^	578.4 ^b^
−30	3.4 ^c^	516.7 ^c^
−35	1.5 ^c^	267.3 ^d^
−40	0.9 ^c^	140.5 ^e^

Results are expressed as the mean of four trials. Mean values with superscripts containing different letters in the same column are significantly different (*p* < 0.05). RoM, rapeseed oil mayonnaise; SoM, soybean oil mayonnaise.

**Table 4:** Variation of destabilization kinetic parameters with freezing temperature for RoM and SoM.

Temperature (°C)	RoM		SoM	
	*k_d_* (min^−1^)	*n*	*k_d_* (min^−1^)	*n*
−20	1.78 × 10^−5^	0.05	1.95 × 10^−6^	0.43
−25	6.40 × 10^−5^	0.14	4.83 × 10^−5^	0.54
−30	4.28 × 10^−4^	1.37	8.59 × 10^−5^	1.35
−35	8.82 × 10^−4^	1.75	3.37 × 10^−4^	0.11
−40	1.28 × 10^−3^	1.25	7.89 × 10^−4^	0.76

Results are expressed as the mean of four trials. RoM, Rapeseed oil mayonnaise; SoM, Soybean oil mayonnaise. *k_d_* (destabilization rate constant) and *n* (order of destabilization) indicated that the destabilization rate constant was dependent on temperature.

**Table 5:** Induction time for generation of fat crystals in RoM and SoM.

Temperature (°C)	RoM Induction Time (min)	SoM Induction Time (min)
−20	360	860
−30	120	710
−40	50	260

**Table 6:** Variation of crystallization kinetic parameters with temperature; Avrami exponent, *n* and crystallization rate constant, *K_c_*.

Temperature (°C)	*n*		*K_c_*	
	RoM	SoM	RoM	SoM
−20	0.78	0.76	9.01 × 10^−3^	7.87 × 10^−5^
−30	0.98	0.94	1.11 × 10^−2^	9.07 × 10^−4^
−40	1.46	1.34	1.14 × 10^−2^	1.87 × 10^−3^

**Table 7:** Variation of crystallization rate constant, *K_c_*, with temperature recalculated using an Avrami exponent, *n*, of 1.

Temperature (°C)	*n*	*K_c_* (min^−1^)			
		RoM	*R* ^2^	SoM	*R* ^2^
−20	1	4.73 × 10^−3^	0.91	3.60 × 10^−5^	0.83
−30	1	1.09 × 10^−2^	0.99	7.68 × 10^−4^	0.99
−40	1	2.80 × 10^−2^	0.90	2.88 × 10^−3^	0.95
